# Performance Analysis and Coefficient Generation Method of Parallel Hammerstein Model Under Underdetermined Condition

**DOI:** 10.3390/s26010183

**Published:** 2025-12-26

**Authors:** Nanzhou Hu, Youyang Xiang, Mingyang Li, Xianglu Li, Jie Tian

**Affiliations:** Institute of Electronic Engineering, China Academy of Engineering Physics, Mianyang 621999, China; hnz596828775@163.com (N.H.);

**Keywords:** parallel Hammerstein model, nonlinearity, memory polynomial model, singular value decomposition, coefficients estimation

## Abstract

Nonlinear signal models are widely used in power amplifier predistortion, full-duplex self-interference cancellation, and other scenarios. The parallel Hammerstein (PH) model is a typical nonlinear signal model, but its serial and parallel hybrid architecture brings difficulties in performance analysis and coefficient estimation. This paper focuses on the performance analysis and coefficient estimation of the PH model for nonlinear systems with memory effects, such as power amplifiers. By comparing the PH model with the memory polynomial (MP) model under identical basis functions, we analyze its performance across varying numbers of parallel branches, nonlinear orders, and memory depths. Using singular value decomposition (SVD), we derive a closed-form expression for the PH model’s performance under underdetermined conditions, establishing its relationship to the non-zero singular values of the MP model’s coefficient matrix. Based on this, we propose a coefficient generation method combining SVD and least squares (LS), which directly computes coefficients and assesses performance during execution. Simulations confirm the method’s effectiveness, showing that selecting branches associated with larger singular values achieves near-optimal performance with reduced complexity.

## 1. Introduction

Radio frequency (RF) nonlinearity is a common problem in sensor communication networks and the nonlinear model finds extensive applications in fields such as power amplifier (PA) predistortion [1,2,3] and full-duplex self-interference (SI) cancellation [4,5,6,7]. Since power amplifiers often incorporate radio frequency components like inductors and capacitors, they can be viewed as nonlinear systems with memory effects. In PA linearization, imperfect modeling directly limits adjacent-channel leakage ratio (ACLR) and error vector magnitude (EVM), degrading link reliability. In full-duplex SI cancellation, residual nonlinear SI—often 20–30 dB stronger than the desired signal—renders linear cancellation ineffective, necessitating precise behavioral models like the PH or MP. Similarly, in active RF sensors (e.g., radar, biomedical tags), nonlinear distortions corrupt sensing fidelity and dynamic range.

Existing nonlinear models for characterizing RF impairments like power amplifiers fall into two categories: those whose output is nonlinear to their coefficients [8,9,10], and those whose output is linear to their coefficients [2,3,4,5,6,7,11]. The former category includes neural network models [8,9], moving average nested models [10], and so on. However, due to the inability to solve model coefficients using classical least squares (LS) estimation methods employed in linear systems, they are less widely applied than the latter category. The Volterra series model, belonging to the latter category, serves as a common tool for characterizing power amplifiers [2,3]. However, the number of Volterra model coefficients increases exponentially with memory depth and nonlinear order, so that it is necessary to intercept the Volterra model to reduce complexity [3].

Memory polynomial (MP) model [4,6,11] and Hammerstein model [12] are widely used in academia and industry, which are represented by Volterra series. The parallel Hammerstein (PH) model, which uses the Hammerstein model in parallel branches, is between the MP model and the Hammerstein model in terms of performance and complexity, and has also been widely studied [12,13,14,15,16,17]. In terms of model performance analysis, the PH model is more complex than the single-level linear combination of the basis function of the MP model due to its serial and parallel hybrid architecture. Although the equivalence between the PH model and the MP model has been discovered when the number of branches is large enough [14], the performance analysis of the PH model is still insufficient when the number of branches is small.

In addition, because the PH model is a mixed structure of serial and parallel, the estimation of its coefficients is relatively complicated. The iterative parametric identification (IPI) methods of the PH model are given in [13,14], but the nonlinear order of each branch is fixed. In [15], the PH model is used to solve the nonlinear RF impairments in OFDM systems, and the nonlinear signal is converted into a linear combination in the frequency domain by using the characteristics of OFDM. The coefficient identification performance based on LS, cross-correlation, and artificial neural networks is compared. For multiple-input and single-output Hammerstein models, a stochastic gradient method is proposed in [16], and its effectiveness is verified by simulation; ref. [17] proposed a chaotic dynamic disturbance sand cat swarm optimization method for parameter identification of PH models, which has advantages in convergence efficiency and identification accuracy. However, the two methods also have the problem of limiting the nonlinear functions on the branches, which essentially reduces the degree of freedom of the model. Beyond model-based approaches, recent works have also explored data-driven optimization techniques, such as proximal policy optimization (PPO) for system-level parameter tuning in communication systems [18]. However, these methods typically require extensive training and lack performance interpretability, whereas our focus remains on analytical, non-iterative coefficient generation grounded in the intrinsic structure of the nonlinear model. At present, the research on the PH model parameter estimation problem, which is generally suitable for general communication systems, is still insufficient.

While both the MP and PH models are widely adopted for characterizing nonlinear systems with memory, a systematic performance comparison under a common basis function set remains lacking, especially under limited model complexity (i.e., a small number of parallel branches). Moreover, existing parameter estimation methods for the PH model, such as IPI, often impose structural constraints (e.g., fixing the nonlinear order per branch) or rely on iterative optimization, which may compromise stability and real-time feasibility.

In this paper, by comparing the performance of the PH model and the MP model under the same basis function set, the performance of the PH model under different numbers of parallel branches, nonlinear orders, and memory depths is analyzed. LS is employed as the baseline coefficient estimator for the MP model because it is non-iterative, computationally efficient, and analytically tractable, providing a fair reference that isolates model–structure effects from estimation algorithm artifacts. Building on the LS-estimated MP coefficient matrix, SVD reveals the intrinsic low-rank structure of typical nonlinear systems, enabling a closed-form, non-iterative coefficient generation (CG) method for the PH model that directly links model complexity to achievable performance. The integration of SVD from the MP coefficient matrix and LS for PH model coefficient generation is dictated by the mathematical equivalence between the PH and MP models under shared basis functions. Consequently, the proposed LS–SVD framework provides both theoretical insight into PH model performance limits and a practical pathway for complexity-aware implementation in embedded sensor communication systems. While prior works have explored IPI [13,14], gradient-based [16], or evolutionary optimization [17] for PH model identification, they share a common limitation: they treat coefficient estimation as a black-box optimization problem, often fixing branch-wise nonlinear orders or imposing structural constraints that reduce model expressiveness. In contrast, the proposed CG method leverages the intrinsic linear-algebraic relationship between the PH and MP models, enabling closed-form, non-iterative coefficient generation guided by singular value analysis. This not only avoids convergence instability but also provides an a priori performance guarantee, features absent in existing heuristic or iterative schemes. The main contributions of this paper are summarized as follows:

- Based on SVD, the closed-form expression of the performance of the PH model with a different number of branches is derived. The results reveal the relationship between the performance of the PH model and the non-zero singular value of the coefficient matrix of the MP model.
- A coefficients generation method of the PH model based on SVD and LS is proposed. The proposed method can directly solve the model coefficients, and obtain the model performance under the current number of branches during the calculation process.

The rest of this paper is organized as follows. Section 2 gives the signal model and performance analysis, where Section 2.1 describes the signal model used in this paper, and Section 2.2 analyzes the performance of the PH model in detail. The proposed method for CG is described in Section 3. Our simulation results and performance analysis are presented in Section 4, and we conclude our work in Section 5.

*Notation:* In this paper, ${\left(\cdot \right)}^{H}$ and ${\left(\cdot \right)}^{T}$ represent the conjugate transpose and transpose of a matrix, respectively. What is more, ${\left(\cdot \right)}^{*}$ indicates the complex conjugate operators, and $E\left[\cdot \right]$ denotes mathematical expectation. $Vec\left(\cdot \right)$ denotes matrix vectorization, while $Vec^{-1}\left(\cdot \right)$ is its inverse operation.

## 2. Signal Model

### 2.1. Review of the Nonlinear Signal Models

In this paper, we consider a nonlinear signal system in which the baseband equivalent input and output signals are expressed as *x* and *y*, respectively. Nonlinear processes in communication systems, such as PA, usually focus on nonlinear effects such as carrier frequency accessories, and Volterra series is a common tool to characterize this process. The baseband equivalent form of the Volterra series model can be given by

(1) yn=fVxn;P,M=∑p=1poddP∑m1=0M⋯∑mp=0Mapm1,m2,⋯,mp∏i=1p+1p+122xn−mi∏j=p+3p+322px*n−mj,

where *P* and *M* represent the highest nonlinear order and the maximum memory depth respectively; $a_{p}\left(m_{1},m_{2},\cdots ,m_{p}\right)$ is the nonlinear model coefficient of the *p*-order cross memory term, also known as the *p*-order Volterra kernel; $m_{i}$ represents the memory delay corresponding to the *i*-th item of the cross memory item; $m_{1},m_{2},\cdots ,m_{p}$, respectively, represent the memory delay corresponding to the first, second,…, *p*-th elements that make up the *p*-order cross memory term.

However, the number of coefficients of the Volterra series model increases exponentially with the memory depth *M* and nonlinear order *P*, and its extremely high complexity makes it almost impossible to be applied in practical engineering. Therefore, it is generally necessary to intercept the Volterra series model to simplify the polynomial model. Considering ignoring the cross-memory terms in the Volterra series model, i.e.,∏i=1p+1p+122xn−mi∏j=p+3p+322px*n−mj,mi≠mj, the MP model can be obtained as

(2) yn=fMPxn;P,M=∑m=0M∑p=1poddPam,pϕm,pxn,

where $\phi_{m,p}\left(x\left(n\right)\right)=x\left(n-m\right){\left|x\left(n-m\right)\right|}^{p-1}$ is the basis function of the memory polynomial, and $a_{m,p}$ is the coefficients of MP model.

Further, by decoupling the nonlinearity and memory effect in the model, the Hammerstein model can be obtained as

(3) yn=fHxn;P,M=∑m=0M−1hm∑p=1poddP−1cpϕm,pxn,

where $c_{p}$ is nonlinear coefficient and $h_{m}$ is coefficient of a finite impulse response.

The number of coefficients of the Hammerstein model is only (M+1)+(P+1)/2 compared with (M+1)(P+1)/2 of the memory polynomial model. Among the intercepted Volterra models, the memory polynomial model and Hammerstein model are the two most common models in the field of signal processing. However, with the development of communication technology, it has been found that the fitting accuracy of the Hammerstein model is slightly insufficient. Therefore, some researchers point out that replacing a single Hammerstein model with a PH model has better general applicability in fitting the nonlinearity of communication systems [13,14], which can be expressed as

(4) yn=fPHxt;P,M,K=∑k=0K−1∑m=0M−1hmk∑p=1poddPcpkϕm,pxn,

where $c_{p}^{k}$ is the nonlinear coefficient and $h_{m}^{k}$ is the coefficient of a finite impulse response at the *k*-th branch.

**Figure 1:**
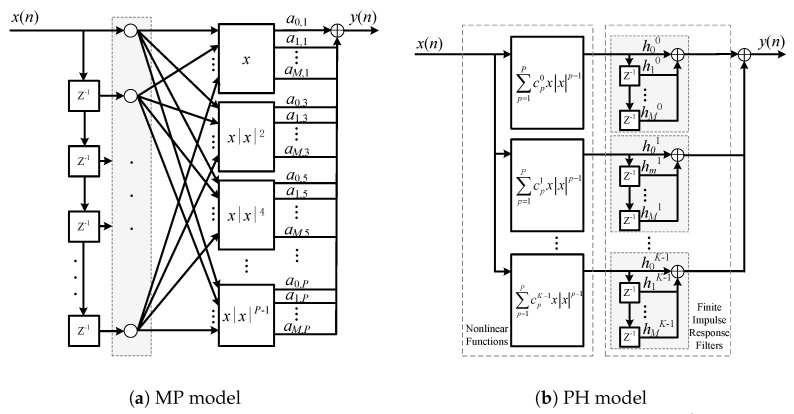
Diagrams of the nonlinear models discussed in this paper. Here, $Z^{-1}$ represents the unit delay element. $x\left(n\right){\left|x\left(n\right)\right|}^{p-1}$ and ∑p=1poddPcpkxnxnp−1 are the delay-removed forms of the basis functions $\phi_{m,p}\left(x\left(n\right)\right)=x\left(n-m\right){\left|x\left(n-m\right)\right|}^{p-1}$ of MP model in Equation (2) and the nonlinear function ∑p=1poddPcpkϕm,pxn of PH model in Equation (4), respectively.

The schematic diagrams of the MP model and the PH model are given in Figure 1. In this paper, we will conduct an in-depth analysis of the performance of the PH model. Specifically, we will discuss how many parallel numbers the PH model can achieve or even exceed the performance of the MP model, and how much performance loss it has compared to the MP model when the parallel number is low. We will show that the latter is actually an underdetermined fitting problem.

### 2.2. Performance Analysis

Before analyzing and discussing the performance of PH model, since this paper focuses on the fitting performance of the model itself, it is assumed that the optimal estimates of the coefficients of the MP model and the PH model can be obtained, regardless of the errors introduced by the coefficient estimation process.

Considering a nonlinear signal, when the nonlinear order *P* and memory depth *M* are infinite, it can be accurately expressed by the Volterra series model, which is given by

(5) yn=∑p=1podd+∞∑m1=0+∞⋯∑mp=0+∞apm1,m2,⋯,mp∏i=1p+1p+122xn−mi∏j=p+3p+322px*n−mj.

We first consider using an MP model with nonlinear order *P* and memory depth *M* to fit the above nonlinear signals, then there is

(6) y^MPn=fMPxn;P,M+εMPn;P,M=∑m=0M∑p=1poddPam,pϕm,pxn+εMPn;P,M

where $\varepsilon_{MP}\left(n;P,M\right)=f_{MP}\left(x\left(n\right);M,P\right)-y\left(n\right)$ is the fitting error. Since the solution of the coefficients of the MP model is a linear optimization problem, by the orthogonality principle [19], the optimal solution of the coefficient $\left\{a_{m,p};m=0,\cdots ,M,p=1,3,\cdots ,P\right\}$ must hold the following equations

(7) $$E\left[\phi_{m,p}\left(x\left(n\right)\right){\varepsilon_{MP}}^{*}\left(n;P,M\right)\right]=0,\forall m=0,\cdots ,M,p=1,3,\cdots ,P.$$

The memory polynomial satisfying the above conditions is the optimal estimate of the nonlinear signal $y\left(n\right)$ under the set of basis functions $\{\phi_{m,p}\left(x\left(n\right)\right);m=0,\cdots ,M,p=1,3,$⋯,P}. Comparing Equations (5) and (6), we can see that the error $\varepsilon_{MP}\left(n;P,M\right)$ is composed of cross memory terms and terms with order higher than *P* or memory depth greater than *M*, which is the part that the basis function set cannot fit. Therefore, we can obtain the following proposition.

**Proposition** **1.** *Considering that the PH model and the MP model have consistent nonlinear order P and memory depth M, then for any number of branches K of the PH model, the fitting performance of the PH model for a nonlinear signal $y\left(n\right)$ will not exceed that of the MP model, i.e.,*

(8) $$E\left[{\left|\varepsilon_{PH}\left(n;P,M\right)\right|}^{2}\right]\geq E\left[{\left|\varepsilon_{MP}\left(n;P,M\right)\right|}^{2}\right]$$

*where*

(9) $$\varepsilon_{PH}\left(n;P,M\right)=f_{PH}\left(x\left(n\right);M,P\right)-y\left(n\right)$$

(10) fPHxt;P,M,K=∑k=0K−1∑m=0M−1hmk∑p=1poddPcpkϕm,pxn

**Proof.**  Based on a PH model with nonlinear order *P* and memory depth *M*, the nonlinear signal y(n) can be estimated by

(11) y^PHn=fPHxt;P,M,K+εPHn;P,M=∑k=0K−1∑m=0M−1hmk∑p=1poddPcpkϕm,pxn+εPHn;P,M

Observing the above equation, it can be found that the set of basis functions of the model is consistent with that of the MP model, both of which are $\left\{\phi_{m,p}\left(x\left(n\right)\right);m=0,\cdots ,M,p=1,3,\cdots ,P\right\}$. From the previous derivation, we can know that based on the MP model, the optimal estimate of $y\left(n\right)$ can be obtained under this set of basis functions. Therefore, under the same set of basis functions, the fitting performance of the PH model will not exceed that of the MP model.    □

**Remark**  **1.**
*Proposition 1 shows that when P and M are consistent, the upper bound of the fitting performance of the PH model is the fitting performance of the MP model. It can be deduced that if the nonlinear order and memory depth of the PH model are smaller than those of the MP model, the performance of the PH model will be inferior. The increase in the number of parallel branches K can only make the performance of the PH model approach the MP model, but can not exceed the latter. In addition, it is also impossible to rely on the increase in K to achieve an accurate fit to the nonlinear signal with a higher order (>P) or a larger memory depth (>M).*

Next, we are concerned about the impact of the number of parallel branches *K* on the fitting performance of the PH model under the condition that the nonlinear order *P* and memory depth *M* are consistent, and how much *K* can ensure that the PH model achieves the fitting performance of the MP model. After derivation, some conclusions are given by Proposition 2.

**Proposition** **2.** *Consider a fitting of a nonlinear signal $y\left(n\right)$ based on the MP model, denoted as $y_{MP}\left(n\right)$, which is given by Equation (6), with a nonlinear order of P and memory depth of M. The coefficient matrix A of the MP model can be decomposed via SVD as*

(12) $$A=U\left[\begin{array}{cc} D & O\\ O & O \end{array}\right]V$$

*where*

(13) $$A=\left[\begin{array}{cccc} a_{0,1} & a_{0,3} & \cdots & a_{0,P}\\ a_{1,1} & a_{1,3} & \cdots & a_{1,P}\\ \vdots & \vdots & \ddots & \vdots \\ a_{M,1} & a_{M,3} & \cdots & a_{M,P} \end{array}\right]$$

*$U=\left[u_{1},u_{2},\cdots ,u_{M+1}\right]$ and V=v1,v3,⋯,vP+1P+122T are unitary matrices of order M+1 and (P+1)/2 respectively, $D=\mathrm{diag}\left(\sigma_{1},\sigma_{2},\cdots ,\sigma_{N}\right)$ is a diagonal matrix composed of non-zero singular values arranged from large to small, i.e., $\sigma_{1}\geq \sigma_{2}\geq \cdots \geq \sigma_{N}$, and N is the number of non-zero singular values.*

*Then, for a PH model with the same nonlinear order P and memory depth M as the MP model, which is given by Equation (11), the following conclusion holds:*

*1.*  *If the number of banches K≥N, the PH model is equivalent to the MP model.*
*2.*  *If K<N, the minimum sum of squares of the coefficients error between the MP model and the PH model is $\sum_{n=K+1}^{N}\sigma_{n}^{2}$, and $h_{k}=\sigma_{k}u_{k},c_{k}=v_{k},k=1,\cdots ,N$ is a set of PH model coefficients that can obtain the minimum error, where*
(14) $$h_{k}={\left[h_{0}^{k},h_{1}^{k},\cdots ,h_{M}^{k}\right]}^{T}$$
(15) $$c_{k}={\left[c_{1}^{k},c_{3}^{k},\cdots ,c_{P}^{k}\right]}^{T}$$

**Proof**.  Since ${\hat{y}}_{MP}\left(n\right)$ is the optimal estimate of $y\left(n\right)$ under the set of basis functions $\left\{\phi_{m,p}\left(x\left(n\right)\right);m=0,\cdots ,M,p=1,3,\cdots ,P\right\}$, the fitting error based on PH model $\varepsilon_{PH}\left(n;P,M\right)$ can be decomposed into the error between PH and MP model $\varepsilon_{PH\to MP}\left(n;P,M\right)$ and the unfit error $\varepsilon_{MP}\left(n;P,M\right)$. Then, the estimated nonlinear signal based on the PH model can be expanded into

(16) y^PHn=fPHxt;P,M,K+εPH→MPn;P,M+εMPn;P,M=∑k=0K−1∑m=0M−1hmk∑p=1poddPcpkϕm,pxn+εPH→MPn;P,M+εMPn;P,M

where

(17) εPH→MPn;P,M=∑k=0K−1∑m=0M−1hmk∑p=1poddPcpkϕm,pxn−∑m=0M∑p=1poddPam,pϕm,pxn=∑m=0M∑p=1poddP∑k=0K−1hmkcpk−am,pϕm,pxn

When $\varepsilon_{PH\to MP}\left(n;P,M\right)=0$, the PH model is equivalent to the MP model, the necessary and sufficient condition of which is

(18) $$\sum_{k=0}^{K-1}h_{m}^{k}c_{p}^{k}-a_{m,p}=0,\forall m=0,\cdots ,M,p=1,3,\cdots ,P$$

Observing the above equation, we can see that when K<minM+1,P+1P+122, the problem is an underdetermined equation so that there is no necessarily a solution, and when K≥minM+1,P+1P+122, the equation must have a set of solutions such that the PH model is equivalent to the MP model.

Next, we discuss the minimum Frobenius norm solution of the equation under the underdetermined condition. Here, the Frobenius norm is equal to the sum of squares of the coefficients error between the MP model and PH model. The problem can be written in matrix form as

(19) $$\min_{h_{k},c_{k}}∥\sum_{k=0}^{K-1}h_{k}{c_{k}}^{T}-A∥$$

Performing singular value decomposition on matrix A yields

(20) $$A=U\left[\begin{array}{cc} D & O\\ O & O \end{array}\right]V=\sum_{n=1}^{N}\sigma_{n}u_{n}{v_{n}}^{T}$$

where $U=\left[u_{1},u_{2},\cdots ,u_{M+1}\right]$ and V=v1,v3,⋯,vP+1P+122T are unitary matrices of order M+1 and (P+1)/2 respectively, $D=\mathrm{diag}\left(\sigma_{1},\sigma_{2},\cdots ,\sigma_{N}\right)$ is a diagonal matrix composed of non-zero singular values arranged from large to small, i.e., $\sigma_{1}\geq \sigma_{2}\geq \cdots \geq \sigma_{N}$, and *N* is the number of non-zero singular values. Then Question (19) can be written as

(21) $$\min_{h_{k},c_{k}}∥\sum_{k=0}^{K-1}h_{k}{c_{k}}^{T}-\sum_{n=1}^{N}\sigma_{n}u_{n}{v_{n}}^{T}∥.$$

Next, the above issues are discussed in two situations.

1. **When** K≥N: Let $h_{k}=\sigma_{k}u_{k},c_{k}=v_{k},k\leq N$ make $\varepsilon_{PH\to MP}\left(n;P,M\right)=0$, that is, a set of solutions can be found such that PH model is equivalent to the MP model.
2. **When**K<N: Since $\left\{u_{n}\right\}$ and $\left\{{v_{n}}^{T}\right\}$ are the row vectors of unitary matrice U and the column vectors of unitary matrice V respectively, we can get
  (22) $$\begin{array}{c} \mathrm{rank}(u_{n}{v_{n}}^{T})=1,\forall n=1,\cdots ,N\\ \mathrm{rank}(u_{i}{v_{i}}^{T}+u_{j}{v_{j}}^{T})=2,\forall i,j=1,\cdots ,N,i\neq j \end{array}$$
  Since $\mathrm{rank}(h_{k}{c_{k}}^{T})=1,\forall k=1,\cdots ,K$, it follows that a parallel branch $h_{k}{c_{k}}^{T}$ can only fit the decomposition term corresponding to one singular value $\sigma_{n}u_{n}{v_{n}}^{T}$. To minimize the error, let $h_{k}=\sigma_{k}u_{k},c_{k}=v_{k},k=1,\cdots ,N$. Thus, the minimum Frobenius norm of the matrix of coefficients error between the MP model and the PH model is
  (23) $$∥\sum_{k=0}^{K-1}h_{k}{c_{k}}^{T}-\sum_{n=1}^{N}\sigma_{n}u_{n}{v_{n}}^{T}∥=∥\sum_{n=K+1}^{N}\sigma_{n}u_{n}{v_{n}}^{T}∥=\sum_{n=K+1}^{N}\sigma_{n}^{2}$$

   □

**Remark** **2.**

- *Proposition 2 establishes the performance difference between the PH model and the MP model when their nonlinear order and memory depth are identical. It holds for any coefficient matrix ***A*** without assumptions on its rank. However, the practical advantage of the proposed approach relies on the empirical observation that ***A*** from real-world nonlinear systems (e.g., power amplifiers) typically exhibits rapid singular value decay. This is because PA nonlinearities are dominated by low-order intermodulation products (e.g., 3rd and 5th order), and memory effects are smooth and short-range, leading to strong correlations among coefficients $a_{m,p}$. Consequently, the non-zero singular values of matrix ***A*** are typically non-uniform, with a few larger singular values accounting for the majority proportion. This enables the PH model to achieve performance approaching that of the MP model using a relatively small number of branches K, which will be discussed in the next section.*
- *On the other hand, the minimum number of branches required to ensure complete equivalence between the PH model and the MP model relates to the number of non-zero singular values in the coefficient matrix of the MP model. In practice, by incorporating certain engineering-based assumptions, further conclusions can be derived, as will be presented in Corollary 1.*

**Corollary** **1.** *Considering the PH and MP model with consistent nonlinear order P and memory depth M, in order to ensure complete equivalence between the two models for any nonlinear signals, the number of branches K of the PH model must satisfy*

(24) K≥minM+1,P+1P+122

**Proof.**  From Proposition 2, it is known that the equivalence of PH and MP models is K>=N. However, the number of non-zero singular values of the coefficient matrix of the MP model cannot be determined because it is necessary to ensure the equivalence of the two models for any nonlinear signal, so it can only be considered in the worst case. Then, it is necessary to satisfy that the number of branches *K* is not less than the maximum number of non-zero singular values *N*, i.e., K≥minM+1,P+1P+122.    □

**Remark** **3.** *In fact, the coefficients of the MP model of nonlinear signals in engineering, such as power amplifier output, have strong randomness, so the row/column vectors of the coefficient matrix often do not satisfy the linear correlation, resulting in the matrix having a maximum rank, i.e., N=rank(A)=minM+1,P+1P+122.*

## 3. Method

**Figure 2:**
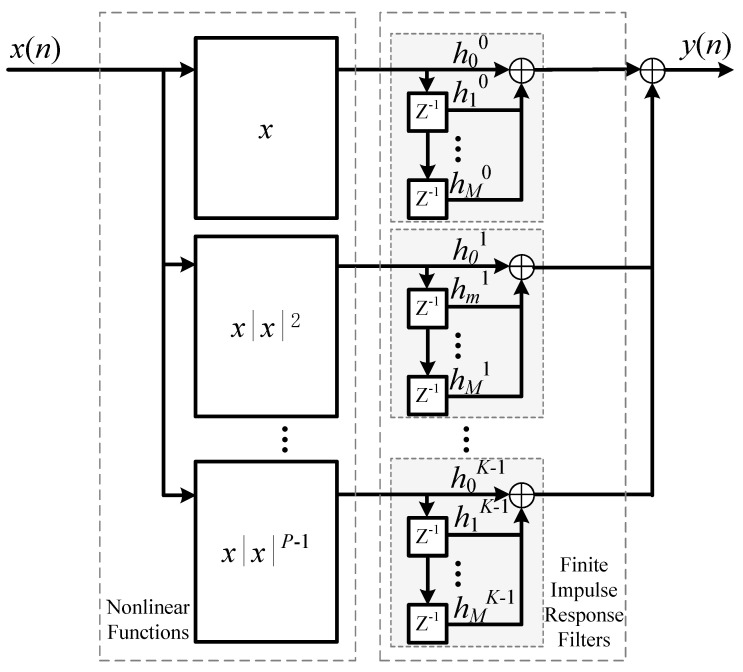
The degenerated PH model.

Despite its structural flexibility, the practical deployment of the PH model is hindered by the lack of a coefficient estimation method that is non-iterative, complexity-aware, and aligned with the intrinsic performance limit revealed by the MP model. To address this deficiency, this section proposes a CG method that directly leverages the SVD-based performance characterization established in Proposition 2. The method is non-iterative, preserves the full degrees of freedom of the PH model, and provides an a priori estimate of achievable accuracy, enabling complexity-aware deployment in real-time sensor and full-duplex communication systems. Compared with the proposed method, gradient or evolution-based methods [16,17] have convergence uncertainty and high computational overhead, making them unsuitable for real-time embedded systems. The IPI methods for the PH model [13,14] restricted the nonlinear function on the *p*-th parallel branch to a single polynomial $x\left(n\right){\left|x\left(n\right)\right|}^{p-1}$. This facilitates coefficient generation but causes the number of parallel branches to determine the highest nonlinear order, as shown in Figure 2. In other words, the PH model degenerates as

(25) yn=f˜PHxt=∑p=1poddP∑m=0M−1hmpcpϕm,pxn

For an MP model with a maximum nonlinear order *P* and maximum memory depth *M*, this degenerated PH model requires at least (P+1)/2 parallel branches to achieve equivalence. This number is greater than min(*M* + 1, (*P* + 1)/2) mentioned in Corollary 1 when M+1≤(P+1)/2. Therefore, this configuration essentially reduces the degrees of freedom of the PH model.

In this section, we propose a coefficients generation (CG) method for the PH model based on the Least Squares (LS) algorithm and SVD. This method imposes no restrictions on the PH model architecture and provides the sum of squared errors between the generated coefficients and the optimal estimates. This guides the determination of the number of branches *K*. When the error is sufficiently small, we can justifiably cease increasing the number of branches, thereby reducing the complexity of the PH model.

Assuming the maximum coefficient error tolerance is λ, the algorithm first obtains the coefficients vector $a={\left[a_{0,1},a_{1,1},\cdots ,a_{M,1},a_{0,2},\cdots ,a_{M,P}\right]}^{T}$ of the MP model through the LS algorithm. Then, by Proposition 1, based on the SVD decomposition of the coefficient matrix A, the branch coefficients of the PH model are gradually generated according to the singular values from large to small. When the sum of the coefficients’ errors is already less than or equal to the tolerance λ, the coefficient generation ends. The error tolerance λ is an absolute threshold on the sum of squared coefficient errors (i.e., Frobenius norm squared of the residual between MP and PH coefficient matrices). Considering that the input signal has been normalized before, a typical choice is $\lambda \in [10^{-4},10^{-2}]$ in practical full-duplex or DPD systems, which corresponds to a residual error power 40-20 dB below the total model energy, sufficient to meet root mean square (RMS) of EVM requirements while avoiding over-parameterization. The exact value can be tuned based on hardware constraints or target cancellation depth. The specific process of the algorithm is listed in Algorithm 1.

The computational complexity of the proposed LS-SVD-CG method is dominated by two steps: (i) the least squares (LS) estimation of the MP coefficient matrix $A\in C^{(M+1)\times (P+1)/2}$, which involves solving a linear system of size $\frac{\left(M+1)(P+1\right)}{2}$ and requires $O(L{\left[\left(M+1\right)\left(P+1\right)/2\right]}^{2})$ operations for *L* data samples; and (ii) the singular value decomposition (SVD) of A, whose complexity is O((M+1)(P+1)/2·min{M+1,(P+1)/2}).
**Algorithm 1** Proposed LS-SVD-CG for the PH model**Require**:
Nonlinear signal y(n), reference signal x(n), maximum coefficient error tolerance λ**Ensure**:
*K*, $\left\{h_{m}^{k},c_{p}^{k}\right\}$ for PH model given by Equation (11)1:$h_{k}=c_{k}=0,k=1,2,\cdots$2:Based on the LS algorithm, the coefficients of the MP model are calculated as$$a={\left(\Phi^{H}\Phi \right)}^{-1}\Phi^{H}y$$
where$$\begin{array}{c} \Phi =\left[\phi_{0,1}\left(x\right),\phi_{1,1}\left(x\right),\cdots ,\phi_{M,1}\left(x\right),\phi_{0,2}\left(x\right),\cdots ,\phi_{M,P}\left(x\right)\right]\\ x={\left[x\left(0\right),x\left(1\right),\cdots ,x\left(L-1\right)\right]}^{T}\\ y={\left[y\left(0\right),y\left(1\right),\cdots ,y\left(L-1\right)\right]}^{T} \end{array}$$3:Rewrite vector a to matrix form $A=\mathrm{Ve}c^{-1}\left(a\right)$4:Performing singular value decomposition on matrix A yields$$A=U\left[\begin{array}{cc} D & O\\ O & O \end{array}\right]V=\sum_{n=1}^{N}\sigma_{n}u_{n}{v_{n}}^{T}$$
where $U=\left[u_{1},u_{2},\cdots ,u_{M+1}\right]$ and V=v1,v3,⋯,vP+1P+122T are unitary matrices of order M+1 and (P+1)/2 respectively, $D=\mathrm{diag}\left(\sigma_{1},\sigma_{2},\cdots ,\sigma_{N}\right)$ is a diagonal matrix composed of non-zero singular values arranged from large to small, i.e., $\sigma_{1}\geq \sigma_{2}\geq \cdots \geq \sigma_{N}$, and *N* is the number of non-zero singular values.5:k=06:**while**$\sum_{n=k+1}^{N}\sigma_{n}^{2}>\lambda$ **do**7:   $h_{k}=\sigma_{k}u_{k},c_{k}=v_{k}$8:   k=k+19:**end while**10:K=k

In practical power amplifier or full-duplex systems (e.g., P≤7, M≤12), the matrix A is small (typically 13×4 or smaller), making both LS and SVD computationally lightweight. More importantly, since the proposed method is non-iterative, its total computational burden is significantly lower than that of iterative approaches such as IPI or gradient-based methods, which require tens to hundreds of iterations per adaptation cycle. This renders LS-SVD-CG highly suitable for online calibration in embedded sensor platforms with stringent latency and resource constraints. For communication systems with stable channel environments (such as full duplex self-interference channels), it is possible to further reduce computational resource consumption based on offline computing and lookup tables.

## 4. Simulation Results

In order to verify the PH model performance analysis results and the proposed coefficients generation method, this section will carry out numerical simulation verification under the system parameters of a full-duplex transceiver as shown in Table 1. Ignoring the cross-memory effect, the nonlinear process of the power amplifier at the transmitter is modeled as an MP model with nonlinear order of P = 5 and memory depth of M = 3, and the polynomial coefficients are given by

$$\begin{array}{c} a_{10}=1.0513+0.0904j,a_{11}=-0.0680-0.0023j,a_{12}=0.0289-0.0054j,\\ a_{30}=-0.0542-0.2900j,a_{31}=0.2234+0.2317j,a_{32}=-0.0621-0.0932j,\\ a_{50}=-0.9657-0.7028j,a_{51}=-0.2451-0.3735j,a_{52}=0.1229+0.1508j. \end{array}$$

The SI channel $h_{SI}$ (n) adopts TDL-A-10ns, which is a channel standard of the third generation partnership project (3GPP) [20]. The specific self-interference channel state information is shown in Table 2.

The system performance is evaluated using the RMS of error vector magnitude (EVM), a standard metric in communication systems whose calculation is usually conducted in the form of RMS, between the true nonlinear output and the model estimate, defined as

(26) $$\begin{array}{c} \mathrm{RMS}=\sqrt{\frac{E\left[|y\left(n\right)-\hat{y}\left(n\right){|}^{2}\right]}{E\left[|y\left(n\right){|}^{2}\right]}}. \end{array}$$

**Figure 3:**
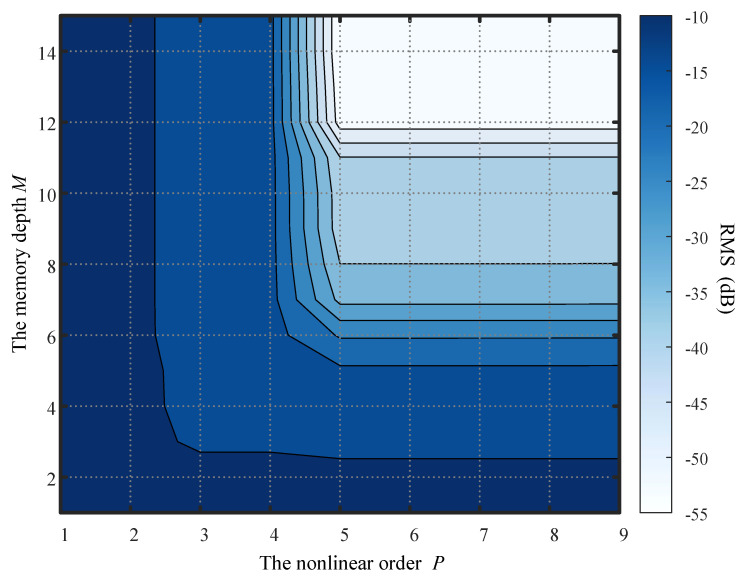
Fitting error based on the MP/PH model under different nonlinear order *P* and memory depth *M*.

Figure 3 shows the RMS of fitting error between the received nonlinear signal and the estimated signal using MP/PH models with different nonlinear order *P* and memory depth *M*. As seen in the figure, when the memory depth M=12 and the nonlinear order P=5, the RMS falls below −55 dB. In practice, a nonlinear order of 5 and a memory depth of 7 already meet the requirements for nonlinear self-interference cancellation in conventional full-duplex systems [6,7]. Although the sum of the power amplifier’s memory depth $M_{pa}=3$ and the channel’s maximum delay $D_{max}=11$ is $M_{pa}+D_{max}=14$, increasing the memory depth beyond 12 in the MP/PH model yields almost no gain. Furthermore, the nonlinear order significantly impacts the RMS, with the maximum performance difference between the models of P=3 and P=5 reaching nearly 40 dB. It indicates the necessity of selecting a nonlinear order capable of covering the main components of the actual nonlinear signal.

**Figure 4:**
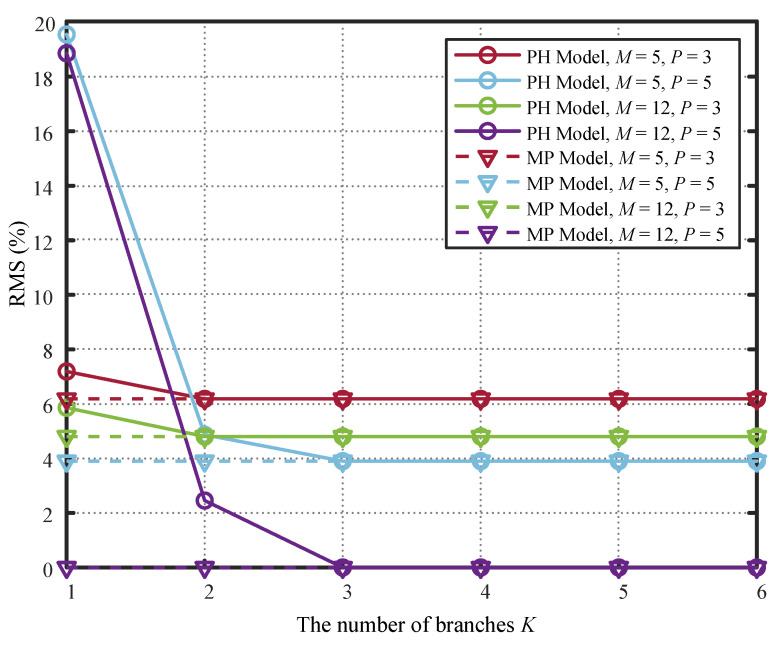
Performance comparison of PH model and MP model under different parameters.

**Figure 5:**
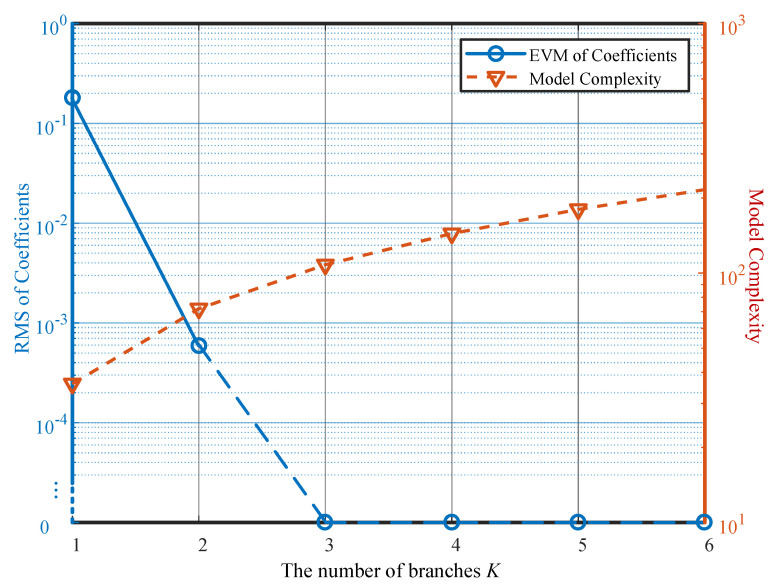
The coefficient fitting error and PH model complexity of the proposed LS-SVD-CG method with respect to the number of branches *K*.

Figure 4 compares the performance of PH models with different numbers of branches *K* against MP models under varying nonlinear orders *P* and memory depths *M*. The performance of the PH model improves progressively with more branches, particularly showing significant gains at low *K*. For the cases where P=3,M=12 and P=5,M=12, the performance curves of the PH model coincide with those of the MP model when the number of branches K reaches 3. Regarding the scenarios of P=3,M=5 and P=5,M=5, since the MP model itself exhibits poor performance due to insufficient nonlinear order P and memory depth *M*, a PH model with only 2 parallel branches suffices to match the MP model’s performance. The results directly confirm Proposition 2 and Corollary 1: for P=5 and M=12, the MP coefficient matrix has only three non-zero singular values (see Figure 5), so K=3 branches suffice for the PH model to achieve exact equivalence with the MP model. Similarly, for M=5, the effective rank is reduced to 2, and K=2 is sufficient. Thus, the required *K* is governed not by the nominal model order, but by the singular spectrum of the underlying system.

**Figure 6:**
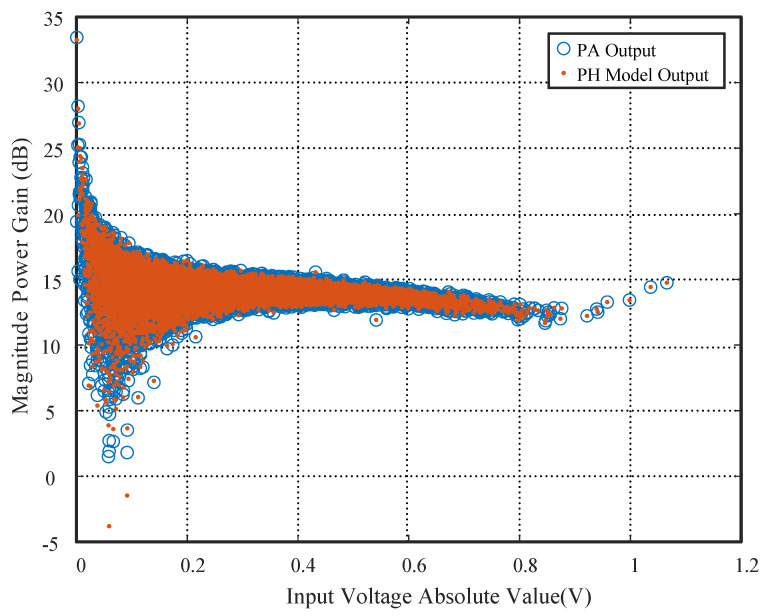
Comparison of power gain transfer functions of power amplifier output and PH model output with nonlinear order P=5, memory depth M=12, and branch number K=3.

Figure 6 shows the power gain transfer functions of the power amplifier output and the PH model output. When P=5, M=12, and K=3, the model output closely matches the PA output, with only minor deviations at low input amplitudes (below 5 dB gain). Since these outliers correspond to low-energy signal samples, their contribution to overall EVM or ACLR is negligible, confirming that K=3 is sufficient for practical system requirements.

Here, we also conducted simulations on the coefficient fitting error and PH model complexity under different numbers of branches *K*. Figure 5 shows that as the number of branches *K* increases, the model complexity continuously grows. However, the coefficient fitting error becomes zero when K≥3, because coefficient matrix A has only three non-zero singular values. Additional parallel branches provide no performance gain for the PH model but only increase its complexity.

**Figure 7:**
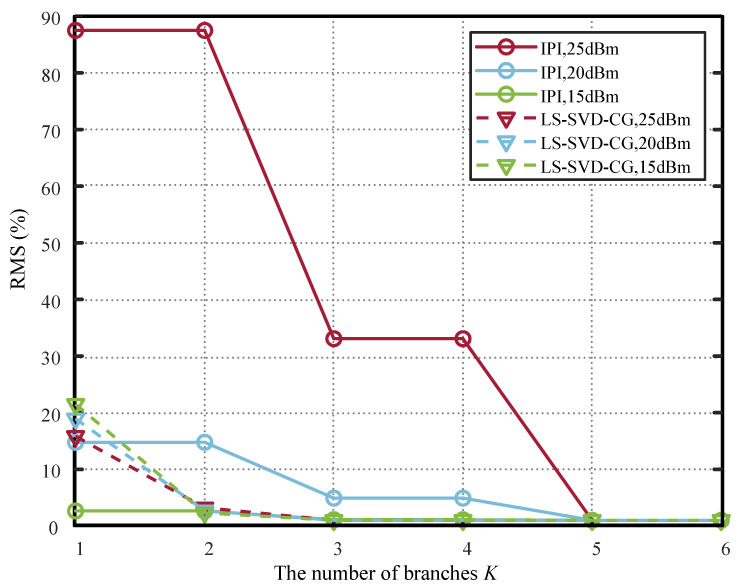
Performance comparison of proposed LS-SVD-CG method and IPI method in [13,14] under different signal power.

Figure 7 compares the performance curves of the PH model based on the proposed LS-SVD-CG method and the IPI method [13,14]. The figure shows that the LS-SVD-CG method underperforms the IPI method when K=1. However, as the parallelism degree increases, the proposed method surpasses the IPI approach until the parallelism degree *K* reaches the highest nonlinearity order P=5. This occurs because LS-SVD-CG performance relates to the number of non-zero singular values in the coefficient matrix A, which is typically less than the maximum nonlinearity order. In contrast, the performance of the IPI method improves as the number of branches *K* increases while simultaneously raising the nonlinearity order. With only one branch, it cancels the strongest linear component in the nonlinear signal, demonstrating superior performance initially. However, it requires K≥P to achieve peak performance. The proposed LS-SVD-CG method performs more stably under different powers, while the IPI method performs worse when the number of branches *K* is small, and the power is large, which is also because of this reason. Simulation results indicate that the proposed method achieves near-upper-limit performance with fewer parallel branches, thereby reducing PH model complexity while ensuring RMS requirements are met.

While Figure 7 shows that the proposed LS-SVD-CG method may exhibit slightly higher RMS error than the IPI method at K=1, its key advantages lie in predictability, stability, and complexity efficiency. First, unlike IPI, which requires iterative optimization and may suffer from convergence issues under high input power (e.g., 25 dBm), the LS-SVD-CG method computes coefficients directly in a single pass, ensuring consistent performance across power levels (as seen in the near-flat LS-SVD-CG curves in Figure 7). This makes it far more suitable for real-time embedded systems where convergence time and algorithmic robustness are critical. Second, the performance of LS-SVD-CG is guided by the singular spectrum of the underlying MP model: it automatically concentrates modeling capacity on the dominant singular components, achieving near-optimal performance once *K* reaches the effective rank (here, K=3, as confirmed by Figure 5). In contrast, IPI incrementally increases nonlinearity order with *K* and only matches MP performance when K≥P=5, leading to unnecessary complexity (e.g., K=4 or 5 branches with full nonlinear orders) for negligible gain. Therefore, the benefit of the proposed method is not merely lower RMS error in all cases, but rather achieving sufficient performance with minimal, a priori-determined model complexity, while avoiding iterative instability, making it particularly advantageous for resource-constrained sensor communication devices.

The simulation results not only validate the theoretical analysis but also carry significant practical implications for real-world system design. First, the observation that only K=3 parallel branches are sufficient to achieve near-MP performance—even under relatively high memory depth (M=12)—implies a substantial reduction in hardware resource consumption when implementing the PH model in embedded digital predistortion (DPD) or self-interference cancellation (SIC) units. In field-programmable gate array (FPGA)- or application-specific integrated circuit (ASIC)-based designs, each parallel branch typically requires dedicated multipliers, adders, and memory elements; reducing *K* from the worst-case bound min(M+1,(P+1)/2)=3 (which coincides here) or from higher values (e.g., K=5 in IPI methods) directly translates to lower silicon area, reduced power dissipation, and decreased latency, critical metrics for battery-powered IoT transceivers or dense full-duplex base stations. Second, the proposed LS-SVD-CG method eliminates iterative training, enabling one-shot coefficient computation during calibration. This avoids the convergence time and potential instability of iterative methods like IPI—especially at high input powers (as shown in Figure 7)—thereby supporting faster online model updates under dynamic operating conditions (e.g., temperature drift or power level changes).

Finally, the ability to predict achievable performance in advance via singular value inspection (i.e., the tail-sum ) allows system architects to make informed trade-offs between model complexity and required cancellation depth before hardware deployment. For instance, if a full-duplex radio only requires −40 dB RMS residual interference, Figure 4 shows that K=2 may suffice, further simplifying the implementation. This predictive capability enhances design flexibility and robustness in resource-constrained sensor communication systems.

## 5. Conclusions

In this work, we have analyzed the performance of the PH model under underdetermined conditions by comparing it with the MP model under identical basis functions. We rigorously established that the PH model’s performance is upper-bounded by that of the MP model, and derived a closed-form expression linking the fitting error to the tail sum of squared singular values of the MP coefficient matrix. This result implies that only a few dominant singular components are typically required to achieve near-optimal modeling accuracy, which was confirmed in simulations: for a PA model with nonlinear order P=5 and memory depth M=12, just three parallel branches suffice to match the MP performance, substantially reducing model complexity.

Building on this insight, we proposed a coefficient generation method combining least squares estimation with singular value decomposition. The method directly computes PH coefficients without iterative tuning and provides an a priori estimate of achievable modeling accuracy, making it well-suited for real-time embedded implementations in sensor communication systems. Its efficiency and stability are particularly advantageous for applications such as full-duplex radios, where low-latency self-interference cancellation under nonlinear RF impairments is critical, as well as for Internet of Things (IoT) transceivers and active RF sensors that demand lightweight yet accurate behavioral models.

Nevertheless, the current approach assumes ideal coefficient estimation from noise-free training data and fixed model orders, which may not hold in practical scenarios with time-varying hardware conditions or limited observation windows. Future work should therefore investigate robust coefficient generation under noisy or data-scarce conditions, adaptive selection of nonlinear order and memory depth based on instantaneous signal or environmental cues, and hardware-efficient deployment of the proposed method in FPGA- or ASIC-based predistortion architectures. Extending the framework to wideband multiple-input multiple-output (MIMO) or envelope-tracking power amplifiers would further broaden its applicability in next-generation sensor networks.

## Figures and Tables

**Table 1:** System parameters.

Modulation	OFDM
Constellation	64 QAM
Number of Subcarriers	1024
Bandwith	100 MHz
Transmitting Power	20 dBm

**Table 2:** SI channel state information.

Delay ($T_{s}$)	Gain
0	0.8122944
1	0.007408687
2	0.085806413
3	0.054045375
5	0.030369523
6	0.009934433
11	0.00014117

## Data Availability

No new data were created or analyzed in this study.
